# Performance analysis of rigorous coupled-wave analysis and its integration in a coupled modeling approach for optical simulation of complete heterojunction silicon solar cells

**DOI:** 10.3762/bjnano.9.216

**Published:** 2018-08-28

**Authors:** Ziga Lokar, Benjamin Lipovsek, Marko Topic, Janez Krc

**Affiliations:** 1 University of Ljubljana, Faculty of Electrical Engineering, Trzaska 25, 1000 Ljubljana, Slovenia

**Keywords:** coupled modeling approach, heterojunction, RCWA, silicon, solar cells

## Abstract

A variety of light management structures have been introduced in solar cells to improve light harvesting and further boost their conversion efficiency. Reliable and accurate simulation tools are required to design and optimize the individual structures and complete devices. In the first part of this paper, we analyze the performance of rigorous coupled-wave analysis (RCWA) for accurate three-dimensional optical simulation of solar cells, in particular heterojunction silicon (HJ Si) solar cells. The structure of HJ Si solar cells consists of thin and thick layers, and additionally, micro- and nano-textures are also introduced to further exploit the potential of light trapping. The RCWA was tested on the front substructure of the solar cell, including the texture, thin passivation and contact layers. Inverted pyramidal textures of different sizes were included in the simulations. The simulations rapidly converge as long as the textures are small (in the (sub)micrometer range), while for larger microscale textures (feature sizes of a few micrometers), this is not the case. Small textures were optimized to decrease the reflectance, and consequently, increase the absorption in the active layers of the solar cell. Decreasing the flat parts of the texture was shown to improve performance. For simulations of structures with microtextures, and for simulations of complete HJ Si cells, we propose a coupled modeling approach (CMA), where the RCWA is coupled with raytracing and the transfer matrix method. By means of CMA and nanotexture optimization, we show the possible benefits of nanotextures at the front interface of HJ Si solar cells, demonstrating a 13.4% improvement in the short-circuit current density with respect to the flat cell and 1.4% with respect to the cell with double-sided random micropyramids. We additionally demonstrate the ability to simulate a combination of nano- and microtextures at a single interface, although the considered structure did not show an improvement over the pyramidal textures.

## Introduction

Light management techniques can be applied to increase the short-circuit current density and consequently the conversion efficiency of solar cells. Such techniques aim to improve the coupling of light into the structure (e.g., antireflective coatings and nanostructures at the front side of solar cells) and the light trapping ability of the structure (e.g., nano- and micrometer size textures for light scattering and refraction). The latter is especially important in solar cells where indirect semiconductors such as silicon (Si) are used as an absorber layer, where the absorption coefficient at the photon energy approaching the value of energy bandgap is small. Furthermore, efficient light management is important in wafer-based Si photovoltaic technologies as the wafers are being thinned down to 150 μm and below. Nowadays different photonic structures (and among them, mostly surface textures of different shapes and sizes) are being tested in solar cells in order to exploit their potential to couple and trap light into solar cells [1–5]. The use of different techniques for the wet and dry etching of Si wafers [6] in combination with thermal or UV nanoimprint lithography [6–7] has opened new potential for design of (nano)textures with superior antireflection, light scattering and trapping properties. Besides the optical properties, proper passivation techniques of textured interfaces are crucial to keep surface recombination velocities as low as possible and thus to maintain the good electrical properties of the device [8–9].

To design and optimize textures applied to the front and/or rear side of solar cells, reliable and accurate optical models implemented in numerical simulation tools are of great importance [10]. The models that enable simulations of thick incoherent and thin coherent layers, including textures of nano-, micro- and several micro-(macro) meter size, are required. Different modeling techniques have been used in simulations of solar cells [11–14], and among them, rigorous coupled-wave analysis (RCWA) has been employed for the optical simulation of thin film or wafer-based solar cells with various textures [2–3,15–17]. However, its applicability, limitations and accuracy in simulation of structures with textures of different types and sizes used in silicon solar cells have not been investigated systematically.

In this paper, we report on three-dimensional optical modeling and simulations applied to a representative of Si-wafer-based technology aiming at low cost production and high conversion efficiency, namely, heterojunction silicon (HJ Si) solar cells [18]. First, we present our optical models and approaches: RCWA and the so-called coupled modeling approach (CMA). The general idea of CMA as a combination of simulators was presented in [19], while its realization by coupling RCWA, raytracing (RT) and the transfer-matrix method (TMM) and its application is presented in this work.

We proceed with the results of the analysis of the applicability and accuracy of the RCWA method for simulation of different textures in nano- and micrometer size, as applied to the front side of a solar cell structure. We quantify the simulation errors with a |Δ*J*_SC_| measure for the various number of sublayers and modes considered in the simulations. The analysis shows that RCWA is an efficient simulation tool for small textures, which is a further verification of the results obtained previously [20]. However, we also show that the method can suffer in terms of accuracy for large (5 μm) textures for what is considered reasonable simulation time (about one day for the complete wavelength range of interest on a typical desktop PC). Additionally, RCWA may have convergence difficulties if systems of equations are large and the layers in the structure have low absorption.

After the applicability and accuracy of RCWA have been successfully tested and analyzed, we apply the RCWA method to optimize inverted-pyramid nanotextures on the front side of the HJ Si solar cell to minimize the reflectivity losses. The CMA approach, where we couple RCWA for nanotextures with RT for thick layers and large textures and the TMM for thin coherent layers, is applied to optimize the complete HJ Si solar cell, which is too complex for any individual simulator due to its size. We show the results of the simulations and discuss the potential and suggestions for improvements in external quantum efficiency (EQE) and short-circuit current density, *J*_SC_, of the HJ Si solar cell by applying different textures (nano, micro and combined nano + micro) to the solar cell structure. As an extension of our previous work presented in [20] and to other coupling approaches such as the OPTOS matrix formulation [21], our CMA was used for the simulation of the solar cell structure including a double (nano + micro) texture.

### Modeling

#### Rigorous coupled-wave analysis method

RCWA, also called the Fourier modal method (FMM), has been widely used in simulations of photovoltaic devices [2–3,15–17], including the structures similar to the ones explored in this paper [3]. It assumes lateral periodicity of the simulated structure.

**Figure 1:**
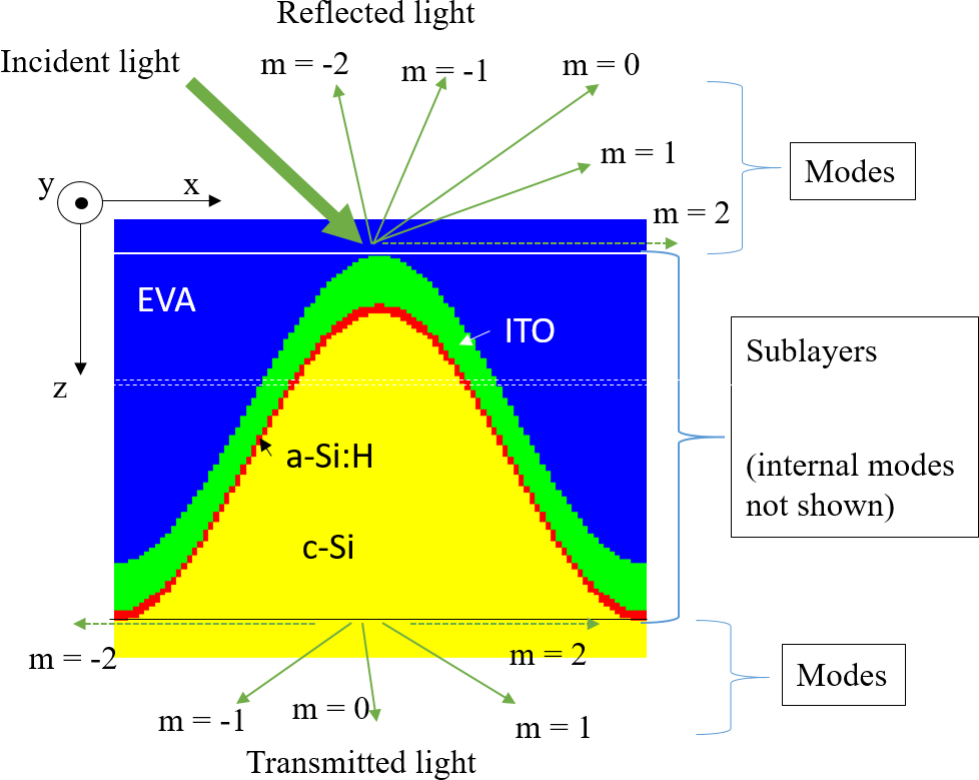
Vertical cross-section of a sliced three-layer structure with texture applied to the bottom layer (sine texture shown in this example). The different 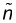 of the layers are indicated by different colors. The structure was in this case sliced to 100 sublayers; a selected one is emphasized by thin dashed horizontal lines. The case for the maximum mode number, *M* = 2, is shown, which corresponds to a total number of modes equal to 25 in 3D simulations (in this 2D cross-section only five modes are depicted (including evanescent modes) for reflected and transmitted light). The internal modes of each sublayer are not shown.

In the RCWA, an analyzed (multilayer) structure is sliced into thin sublayers [22] (see Figure 1) as an example of a multilayer structure with applied texture. Inside a sublayer, materials with different complex refractive indices 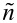 are involved in lateral directions (*x*, *y*). No vertical dependence (*z*) of 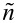 is assumed inside a sublayer, while lateral changes of 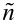 are considered to be abrupt. This results in a staircase approximation of 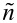. While lateral periodicity of the simulated structure is assumed in RCWA, random textures can be simulated by including a sufficient segment of the structure to form a pseudo-periodic simulation domain, where the statistical parameters of the random roughness are still well represented [3]. Spatial 2D discrete Fourier transform of 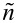 staircase distributions is applied to all (*N*) sublayers, obtaining a discrete power spectrum of 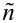 distribution for each sublayer. These Fourier components are then combined with wavevectors in a matrix describing the propagation of light inside each sublayer separately. The matrix size depends on the number of modes considered. Based on this matrix, complex vectors of the electric and magnetic field, ***E*** and ***H***, inside each sublayer can be defined at the end of the calculation. Eigenvectors of the matrix define lateral dependence of ***E*** and ***H***, while eigenvalues describe their vertical dependence. Finally, boundary conditions at the interfaces of sublayers are defined considering that tangential components of ***E*** and ***H*** need to be conserved for conservation of momentum. When solving the system, an S-matrix algorithm is typically used in the RCWA method to couple equations between different sublayers [23–25]. For the purpose of further integration and adaptation, we developed and verified our own RCWA simulation tool in MATLAB, following the physics described above.

To carry out reliable and accurate simulations of solar cell structures with RCWA, it is of prime importance to study the role of input settings first. In our analysis, we focus on the role of the number of sublayers and the number of modes used in simulations. A higher number of sublayers improves the geometrical description of the structure. The maximum mode number, *M*, defines where the discrete Fourier spectrum of 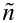 is cut and at the same time how many diffraction modes (directions) of light we consider in our calculation (some might also be evanescent). A higher number of modes leads to both a better description of the actual light propagation and diffraction, as well as improved structure accuracy (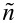 distribution) by taking more Fourier components. However, it also leads to an increase of the size of the system of equations, so it is desirable to use as low number of modes as possible, while maintaining suitable accuracy of simulations. The results of the analysis are shown in the section “RCWA accuracy analysis of partial cell structure” for selected realistic nano- and microtextures for the case of a HJ Si solar cell.

### Integration of RCWA into the coupled modeling approach (CMA)

**Figure 2:**
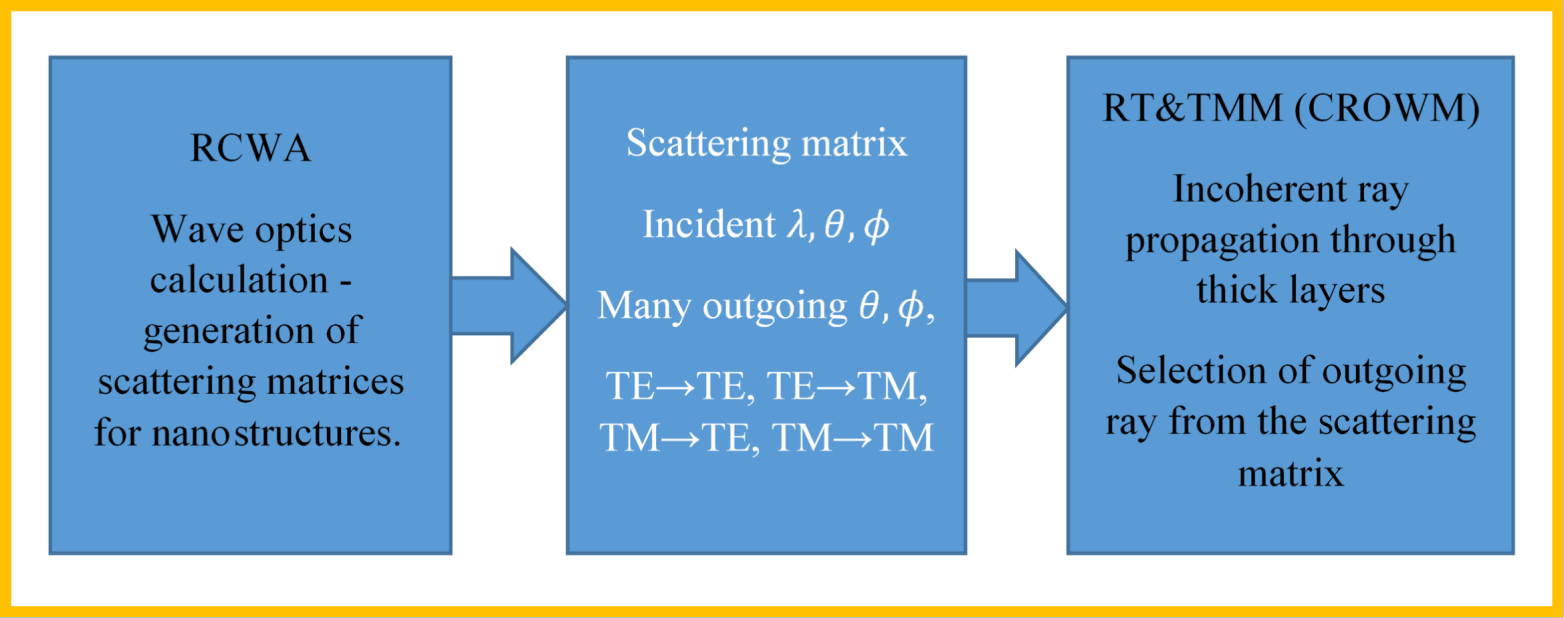
Principle of the coupled modeling approach (CMA). RCWA is applied to the parts of the structure where nanotextures are present to produce scattering matrices. These matrices are an input for the RT and TMM part of the simulator. By applying iterative coupling, the optical situation in the region of nanostructures, microstructures, thin and thick layers can be simulated in an effective and accurate way.

The next simulation approach that we will use in our simulation study enables simulations of a complete HJ Si solar cell structure, including either nano-, micro-, or combined (nano + micro) textures in the same structure. Furthermore, thin coherent and thick incoherent layers are included. The realization of high efficiency solar cells requires the capability of modeling such optical structures [26]. We successfully coupled 3D RCWA with 3D raytracing and transfer matrix formalism (TMM) and call the approach the coupled modeling approach (CMA) [27]. The experimental validation of the RCWA and CMA has been performed. Besides comparing simulation results to the results obtained with other simulators (such as FEM as presented), experimental verification of the RCWA and CMA has been performed. The results will be published elsewhere, whereby the focus of this paper is firstly a detailed analysis of RCWA simulation applicability and accuracy, and secondly to use RCWA for optimization of the inverted-pyramid nanotexture, and thirdly, integration in CMA and applicability of CMA for simulation of a fully encapsulated silicon heterojunction solar cell. For the combination of RT and TMM we employed the previously developed optical simulator CROWM [13,28–29], which was previously tested and experimentally verified on different solar cell structures, including thick macrotextured layers (RT simulation) and thin-film layers (TMM simulation). Whereas RCWA is used for detailed description of optical situation in thin nanometer-textured stacks, raytracing and TMM are utilized to define the optical situation in the region of micro- or macrotextured thick or thin layers. The incoherent nature of light in thick layers is assured by the RT algorithm, while coherent RCWA requires wavelength averaging to eliminate interference fringes. The principle of the presented CMA is schematically shown in Figure 2.

Both plane waves of RCWA and TMM as well as geometric rays of the RT method have well defined wavelength, angles of propagation (θ and 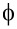 correspond to polar and azimuth angle, respectively) and intensities. The intensities are divided into transversal electric (TE) and transversal magnetic (TM) polarization components. The RCWA waves can be simply transformed into rays and back, as the phase is not needed when propagating in incoherent parts of the cell. This makes the combination of the methods very suitable to couple, as there is no need for additional transformations, unlike the combinations of raytracing with other methods. However, the phase can also be considered in the presented CMA if, e.g., only thin coherent layers would be coupled. One should note that in general, the polarization of a wave with respect to the normal of the interface can change from TE to TM or vice versa. This is unlike in (locally) flat interfaces considered with ray optics or TMM, where local TE and TM polarization persist after reflection or refraction. This difference in 3D wave simulations is caused by the diffracted waves, which may not propagate in the same plane as the incident wave. Significant errors are produced if polarization changes are not considered properly.

In CMA simulation, RCWA results for the assigned sub-structure are calculated in advance for various predefined discrete incident angles. For discretized directions and wavelengths, a scattering matrix of outgoing waves (modes) is generated – an individual scattering matrix is generated per each discretized direction. Then this matrix is considered in iterative coupling of RCWA part with RT&TMM. In case of presented simulations, the matrices were calculated for each 5° polar incident angle θ and 15° azimuth incident angle 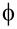, for both TE and TM polarization, for each discrete wavelength λ in the range from 350 nm to 1200 nm in steps of 10 nm. Random selection of waves was used, as given by the intensity of light in a particular direction in the scattering matrix, since the number of applied and reflected/transmitted waves was sufficiently large for this type of approach. In comparison of the presented CMA to the OPTOS simulation tool [29] which generates scattering matrices for all layers and stacks them together, we are able to trace rays throughout the structure at their exact angles and positions, which gives us greater versatility in structure we are able to consider. For example, we are able to simulate the previously mentioned nanotexture + microtexture on the same interface (see section Simulations of an encapsulated solar cell with textures). Additionally, similar to the approach of Rothemund et al. [30], we are also able to perform RCWA calculation of each individual ray at exact angle and polarization if even greater accuracy is desired; however, at the expense of longer simulation times. The CMA enables simulation of single and multi-junction solar cells and photovoltaic modules, such as perovskite-crystalline silicon tandem solar cells [31] including nano, micro and combined textures. In this paper, we focus only on heterojuction silicon solar cells.

CMA simulations were performed for different discretization steps in the polar and azimuth angle to determine the proper input settings for the simulations. The simulation results of the considered structures indicated that for a 5° discretization step of the polar angle and 15° step for of the azimuth angle, only small differences in the results were observed as compared to 1° discretization steps for selected wavelengths. Increasing the discretization step to 10° for the polar angle or 90° for the azimuth angle leads to noticeable simulation errors. Applying the mentioned discretization of 5° and 15° leads to 75 times less simulations than simulating with 1° discretization and was thus used to speed up simulations for the complete wavelength range of interest. Even these parameters lead to approximately 25,000 RCWA simulations for the complete wavelength range of interest, resulting in a total of approximately three days for a simulation of the complete wavelength spectrum on a desktop PC. The same set of RCWA-generated scattering matrices with given nanotexture was then used for all presented CMA simulations of the given structure, leading to significant timesaving.

### Analyzed structure and textures

**Figure 3:**
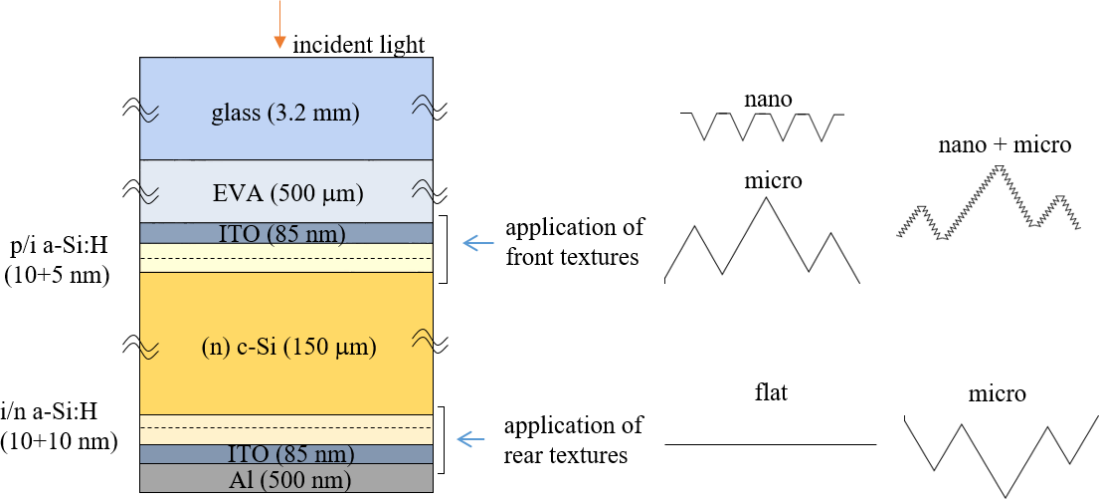
Schematic representation of the simulated HJ Si solar cell structure including illustration of the front and rear textures.

We simulated a realistic structure of an n-type HJ Si solar cell, including front glass and ethylene-vinyl acetate (EVA) encapsulation (Figure 3).

The front of the basic solar cell structure consists of transparent conductive oxide (e.g., indium tin oxide (ITO)), a thin p-doped and intrinsic amorphous silicon (a-Si:H) layer for electrical passivation, a slightly n-doped crystalline Si (c-Si) wafer (absorber), and an intrinsic and n-doped a-Si:H stack; the rear consists of an ITO/Al contact. The textures can be applied to the front and/or rear part of the wafer. In our model, thin layers follow the applied wafer textures. The complex refractive indices of the layers used as input for optical simulations were taken from the PV Lighthouse database [32] and correspond to measurements of realistic layers [18,33–35].

In our analysis, two types of textures were included and applied to either the front or rear interfaces: periodic inverted pyramids or random pyramids. We intentionally focus on the two textures that are commonly applied in HJ Si solar cells. The first one can be experimentally realized on the nanometer scale by UV nanoimprint lithography (NIL) in combination with dry and wet etching of the wafer [6]. The second, the random pyramid texture, is typically used as a microtexture in c-Si solar cells and can be obtained by wet etching with KOH [36].

**Figure 4:**
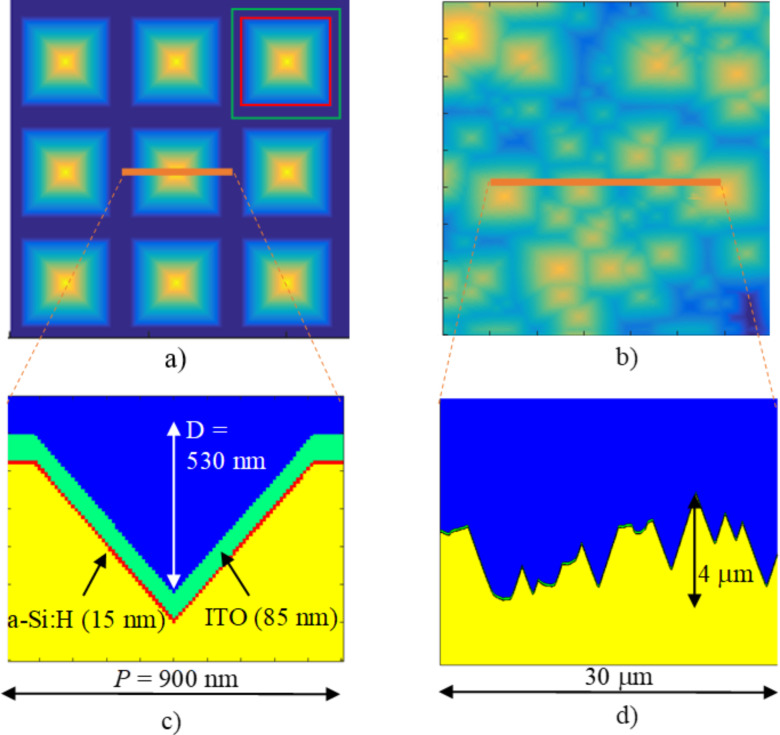
The top and cross-sectional views of the simulated partial structures, applied to the front part of the solar cell. (a, b) simulated top views of inverted pyramid and random pyramid textures. (c, d) Corresponding cross-sectional views, including thin layers as present in the front part of the analyzed solar cell (not seen in (d)). The pixilation observable in the thin layers in c) is a result of sublayer discretization in RCWA (100 equivalently thick sublayers for the texture are shown). In a), the area of the pyramid is marked with red square, while area of the unit cell is marked with a green square. The PF factor, defined by the ratio of these two areas, is 0.7.

In Figure 4 simulated top views and cross-sectional profiles of the two textures are presented, in this case applied to the front part of the analyzed solar cell. The corresponding front thin layers are indicated by different colors. In Figure 4a,c the periodic inverted nanopyramid texture is shown for the case of a period *P* = 900 nm and depth *D* = 530 nm, giving an aspect ratio *D/P* of 0.59. Besides these parameters, the pyramid fraction (PF) is defined as the ratio between the area of the inverted pyramid (red square) and the area of the unit cell (green square) and is 0.7. The depth is dependent on the PF as the pyramid facets are defined by the slow-etching crystallographic plane 111 [6]. In Figure 4b,d the top view of the random microtexture is shown for lateral range of 40 × 40 μm^2^, and a 30 μm long cross-section is shown. In simulations, a random texture from an AFM scan was mirrored across the *x* and *y* axis, resulting in an 80 × 80 μm^2^ so-called pseudo-periodic texture used in simulations. A similar approach was also taken in [3]. The micropyramid faces are also defined by the slow-etching 111 crystallographic plane, leading to the same 54.7° angle. The vertical span of the random micropyramids is 9.5 μm, while the correlation length is 3.1 μm.

Figure 4 shows the textures at the front part of the solar cell, although in the analysis of the complete solar cell (see the section “Simulations of an encapsulated solar cell with textures”) the random microtexture will also be applied to the rear side of the solar cell. Moreover, the combination of both textures (nano + micro) will be applied to the front interfaces. The combination assumes a nanotexture (including thin layers) superimposed on the random micropyramid texture in the direction normal to the random micropyramids. The top view corresponds to the top view of random micropyramids in Figure 4b. In the RCWA analysis, only the inverted pyramid texture is investigated, while the pseudo-random microtexture will be simulated using CROWM. The CMA will be used for all the simulations that contain nanotexture on the solar cell level. The simulated structure combinations are summarized in Table 1.

**Table 1:** Analyzed structures.

	layers (Figure 3)	textures (Figure 4)

partial structure (front part)	EVA (incident medium)/ITO/a-Si:H/c-Si (outgoing medium)	1. inverted pyramids, *P* = 900 nm, *D* = 530 nm, PF 0.7 2. inverted pyramids, *P* = 1800 and 5000 nm, *D* = 1060 nm and 2940 nm, PF 0.7 3. inverted pyramids, P = 900 nm, *D* = 530–570 nm, PF 0.7–1
full cell	air (incident medium)/EVA/ITO/a-Si:H/c-Si/ a-Si:H/ITO/Ag/air (outgoing medium)	front (EVA→c-Si): 1. inverted pyramids (*P* = 900 nm, *D* = 730 nm, PF 1) 2. microtexture (median height 5 μm, 6 x 10^4^ pyramids/mm) 3. microtexture + inverted pyramids superimposed (1+2) rear (c-Si→Ag): 1. flat 2. microtexture

The partial structures were analyzed in the sections “Nanoscale inverted pyramids” (texture 1), “Microscale inverted pyramids” (textures 2) and “Optimization of the nanopyramids with RCWA” (textures 3) with RCWA (also FEM and RT/TMM in comparison). Smaller inverted pyramids (textures 1 and 3) were analyzed at an angle of incidence of 0° and 45°, while larger inverted pyramids (texture 2) were analyzed only for normal incidenct light. Full cell simulations were performed in the section “Simulations of an encapsulated solar cell with textures” using RCWA for generation of scattering matrices for nanotextures, while RT/TMM was used for the simulation of microtextures and propagation through thick (incoherent) layers. All full cell simulations were performed at 0° light incidence.

## Results and Discussion

### RCWA accuracy analysis of partial cell structure

The applicability and accuracy of RCWA for solar cell simulations was tested first on a simpler structure – the front part of the analyzed solar cell (EVA/ITO/p/i a-si:H/c-Si) with the inverted pyramid texture (as shown in Figure 4). With this we avoid inclusion of the rear texture at the same time and the incoherent c-Si layer (a c-Si wafer was considered in these simulations only as infinite medium in transmission; the same holds for EVA in reflection direction). In the analysis, we include the inverted pyramid nano- and microscale textures and check the accuracy of simulations. In particular, the effect of the number of sublayers and number of modes used in RCWA simulations was analyzed for two different angles of incidence (0° and 45°). This is an important step before applying the RCWA simulation in the optimization of the texture (see the section “Optimization of the nanopyramids with RCWA”). Normal incident light analysis is a common case in measurements and an important case in outdoor conditions; therefore, we consider it as an important case for verification and optimizations [37]. The incident angle of 45° has been chosen as a representative of oblique illumination. According to the results of additional simulations, the inclusion of other angles would lead to similar conclusions.

Increasing the number of sublayers requires solving more systems of equations and thus the computational time grows linearly. Increasing number of modes greatly increases the size of the system of equations and is especially demanding for both memory and computational time, with approximate time dependence on the order of M^5^ [38]. Thus, simulations of structures requiring many modes or sublayers may quickly become unfeasible for efficient simulation and optimization of solar cell structures. On the other hand, smaller number of sublayers and modes may lead to inaccuracies of results.

In next subsections we present simulation results on total reflectance, *R*, in the EVA medium of the analyzed partial structure. Besides comparison of wavelength-dependent *R* for different numbers of sublayers and modes used in simulations, we introduce another quantitative measure that highlights the deviations of the different simulations. As the *J*_SC_ of the solar cell is the most important quantity related to optical confinement in solar cells, we calculated an absolute difference (error) in *J*_SC_ reflectance loss, |Δ*J*_SC_|, from deviations in *R*, as given in Equation 1 as

[1]
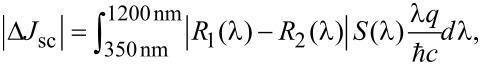


where *q* is the elementary charge, 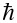 is the reduced Planck constant and *c* is the speed of light. This parameter is defined as the absolute value of the reflectance difference, which is weighted by the AM1.5g solar spectrum *S*(λ). The *R*_2_(λ) is defined as the most accurate simulation obtained, which was ten modes and 300 sublayers for all the cases presented. We validated these parameters with a 20 mode, 1000 sublayer simulation at the reflectance peak, but full analysis at such accuracy is time prohibitive. *R*_1_(λ) is defined as the reflectance of the tested simulation.

In this study, the validation of RCWA is performed by comparison to simulation results obtained by applying different simulation techniques (finite element method (FEM) for nanotextures and RT/TMM for microtextures). In this way, measurement uncertainties of samples are avoided and internal quantities, such as the internal reflectance in EVA, can be determined and compared. The FEM and RT simulators used here for reference have been experimentally validated [37], whereas detailed experimental validation of RCWA will be published elsewhere.

### Nanoscale inverted pyramids

The period and height, as well as the resulting aspect ratio and pyramid fraction of the analyzed nanoscale inverted pyramids were chosen to be *P* = 900 nm and *D* = 530 nm in this nanoscale section. They had an aspect ratio of *D*/*P* = 0.59 and PF = 0.7 (the same as depicted in Figure 4). Textures with *P* and *D* in this range were previously identified for this particular type of solar cell as one of the most efficient light management structures [6]. The analyzed structures also contain thin layers – 85 nm thick ITO and 15 nm thick a-Si:H. The total height of the simulated structure is *H* = 630 nm.

**Figure 5:**
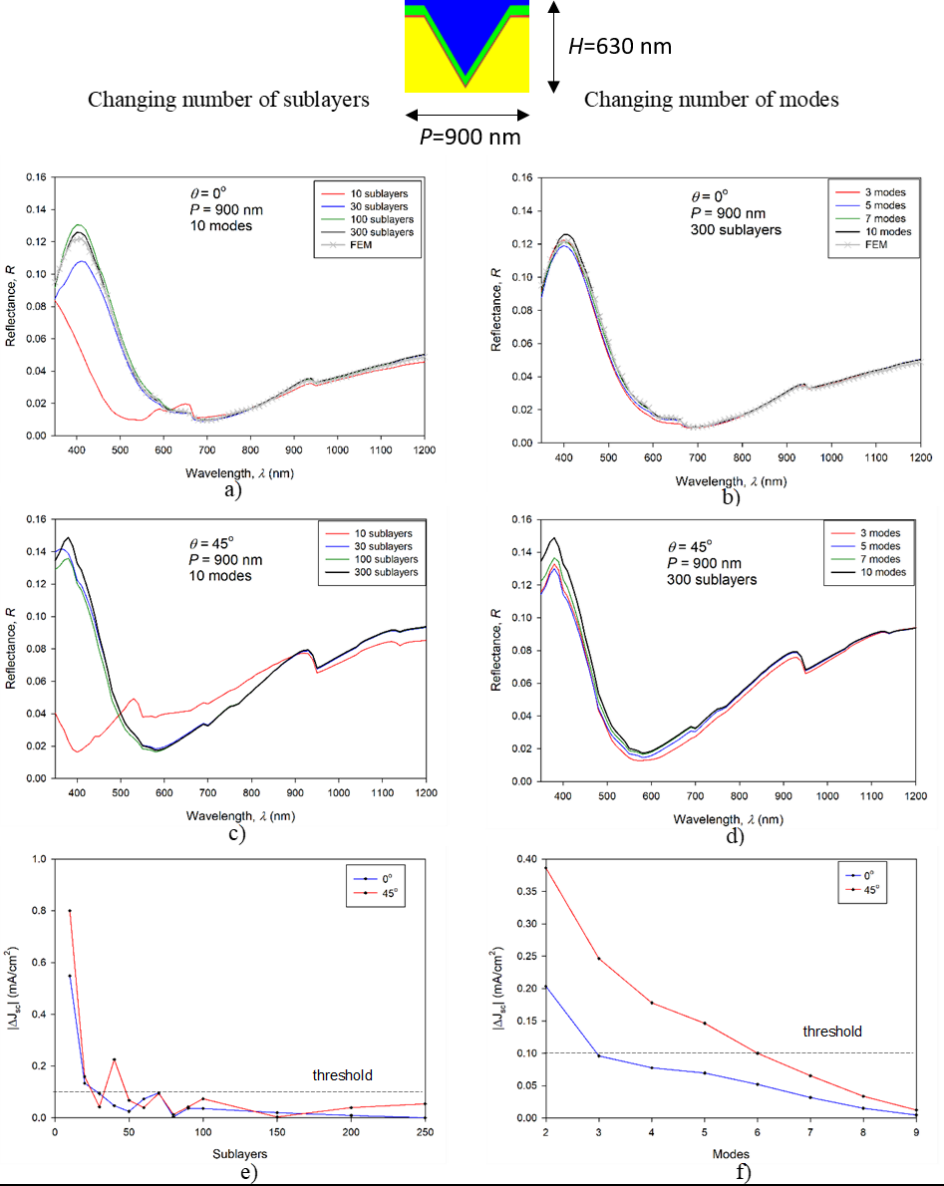
Analysis of the RCWA convergence for the nanoscale textures. All graphs on the left hand side correspond to the results of the variation in the number of sublayers with a fixed number of modes (ten modes) whereas the right hand side graphs represent the results of the variation in the number of mode with a fixed number of sublayers (300 sublayers). The top two graphs (a, b) correspond to an incident angle of 0° whereas the middle two (c, d) are for the incident angle of 45°. The bottom two graphs (e, f) quantify deviations between RCWA results using the |Δ*J*_SC_| measure.

The plots on the left side of Figure 5 show the effects of the variation in the number of sublayers. The plots on the right side demonstrate the effects of the variation of the number of modes. The top two graphs of Figure 5 (a) and (b) correspond to the reflectance of the substructure with inverted pyramid textures and vertical light incidence, the middle two graphs (c) and (d) present the reflectance of the same substructure at 45° light incidence, whereas the bottom two graphs (e) and (f) are the corresponding |Δ*J*_SC_| values for RCWA simulations with different numbers of sublayers and modes compared to the reference simulation.

A general observation from the reflectance curves (Figure 5) is that the vertical light incidence (0°) leads to somewhat lower reflectance than other angles of incidence (shown for 45°). For validation of the reflectance behavior, simulations obtained by FEM [39] are added for the case of the inverted pyramid texture for normal incidence light. 45° light incidence prevents the use of all symmetries. This angle leads to larger simulation volumes and large simulation errors in FEM simulations. It must be mentioned that this simulation was not taken as a reference for the accuracy study. Its purpose is to additionally validate the wavelength dependence of the reflectance obtained by RCWA.

Furthermore, we analyze the effects of the number of sublayers used in the RCWA simulation. The results in Figure 5a,c suggest that for good convergence towards a steady solution, 30 sublayers are sufficient for both angles of incidence, at least for wavelengths above 450 nm. The errors in *J*_SC_ as a function of the number of sublayers (Figure 5e) indicate a lower |Δ*J*_SC_| for the case of normal incident light. 300 sublayers were used as the reference (most accurate) simulation. For 30 or more sublayers, the simulated |Δ*J*_SC_| drops below the chosen threshold line of 0.1 mA/cm^2^ for 0° light incidence, while 50 sublayers are required for 45° incidence. This threshold corresponds to 0.27% of the total *J*_SC_ reflectance loss (36.87 mA/cm^2^) of the structure with inverted pyramidal nanotexture on the front side and flat rear side.

We proceed with the analysis of the number of modes used in RCWA simulations (Figure 5b,d). The first observation is that even three modes are sufficient for predicting the correct reflectance trends for both the vertical and non-vertical light incidence. The convergence of non-vertical incidence is again a bit worse than for the vertical incidence, although the differences are small from 500 nm onwards. The errors in *J*_SC_ as a function of the number of sublayers show that the |*J*_SC_| error corresponding to case of normal incident light drops below the threshold of 0.1 mA/cm^2^ with three modes (ten modes were used as the reference), while in case of non-normal incident light, at least six modes are required to meet this threshold.

When comparing the |Δ*J*_SC_| plots corresponding to the variation in the number of sublayers (Figure 5e) and number of modes (Figure 5f), we generally observe a smaller effect for the changing number of modes (note the different scales). 50 sublayers and six modes were found to be sufficient for both normal incident light as well as at 45° incidence, considering the chosen 0.1 mA/cm^2^ threshold. These settings were considered as the minimal required values in our further simulations of structures with nanotexture. In application of RCWA to optimize the inverted pyramid nanotextures (see section “Optimization of the nanopyramids with RCWA”), we used 300 sublayers and ten modes. In CMA simulations (see section “Simulations of an encapsulated solar cell with textures”), we used 100 sublayers and 5 modes in the RCWA part as the initial angle of incidence of light is perpendicular to the surface (0°), and we tested the parameters to be sufficient for good accuracy.

### Microscale inverted pyramids

The lateral and vertical dimensions of the inverted pyramid texture were extended here by the same factor compared to textures analyzed in the previous section, while maintaining a constant thin-layer thickness. The same convergence analysis was carried out for the textures with *P* = 1800 nm and 5000 nm to detect possible limitations of the RCWA method with respect to feature size. The same number of modes and sublayers were used to make the comparison at equal simulation times (approximately one day for the most accurate case of ten modes and 300 sublayers on a typical desktop PC). The first period was selected as the double of the *P* = 900 nm nanotexture, while the second value was selected to approach the size of an individual micropyramid of the given random texture. The aspect ratio *D*/*P* was kept constant at 0.59 with respect to the nanotextures, resulting in *D* = 1060 nm (*P* = 1800 nm) and 2940 nm (*P* = 5000 nm). Likewise, the PF was also kept constant at 0.7, as was the thickness of ITO (85 nm) and a-Si:H (15 nm). For validation we added in this case simulations with RT/TMM (using the simulator CROWM) for the texture with *P* = 5000 nm. Such large features can be tackled with geometrical optics and are too large for FEM simulation, which was used for simulation of the nanotextures. The results corresponding to normal incident light are presented here.

**Figure 6:**
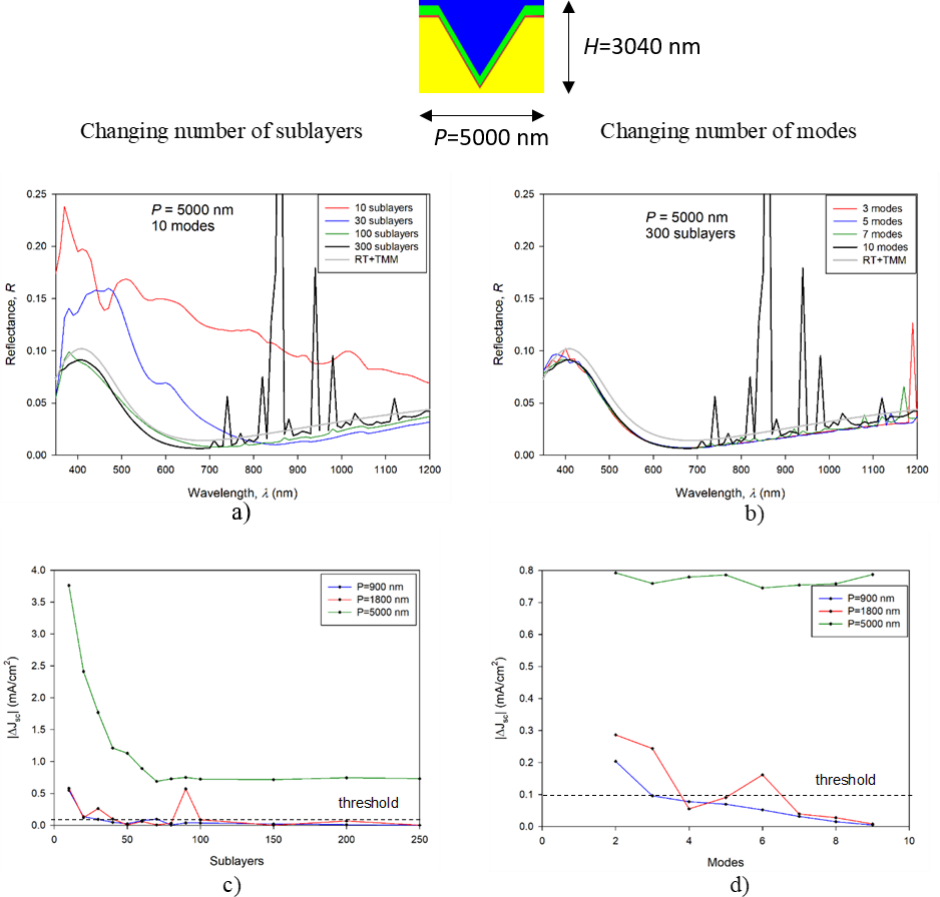
Analysis of RCWA convergence for micrometer-sized textures. Left hand side graphs correspond to the results of variation of the number of sublayers with a fixed number of modes, while the right hand side graphs correspond to the results of variation of the number of modes with a fixed number of sublayers. The top two graphs (a, b) show visual pyramidal texture convergence with number of sublayers and modes. Graphs (c) and (d) quantify the difference between the RCWA results using the |Δ*J*_SC_| measure.

The reflectance curves in Figure 6a,b correspond only to the largest texture (*P* = 5000 nm, *H* = 2950 nm), whereas the |Δ*J*_SC_| results in Figure 6c,d are presented for the textures with *P* = 900 nm (from the previous analysis), 1800 nm and 5000 nm.

The results for the texture with *P* = 5000 nm indicate a much larger effect of the number of sublayers on the reflectance curves (Figure 6a), compared to the texture with *P* = 900 nm (Figure 5a). Smaller deviations are observed with respect to the number of modes (Figure 6b). Many artefacts occur for ten modes and 300 sublayers with some present for lower numbers of modes as well. We assign these artefacts to numerical errors in the RCWA simulations. In particular, with an increasing number of sublayers and modes, the system of equations becomes rank deficient either due to numerical difficulties or due to particular match between period and wavelength [22]. Rank deficiency leads to inaccurate results, as can be seen with the artefacts, or even results in no solution. The additional simulations of structures with macrotextures showed that the system becomes more stable if materials with higher absorption are used, as the numerical artefacts cannot propagate throughout the layers.

Furthermore, the results do not converge to the final solution even if the number of sublayers is increased to 300, as they did in the case of the substructure with *P* = 900 nm. The general trend of the results shows a gradual approach towards the shape of the curve obtained by RT/TMM results, while the offset remains even at 300 sublayers. This offset is not assigned to any systematic error in RCWA or RT/TMM simulations. A single wavelength simulation of the structure sliced to 1000 sublayers was performed at λ = 400 nm, obtaining 1% error in *R* (λ = 400 nm) compared to RT/TMM, whereas in the simulation with 300 sublayers, this error was 10%. A further increase in the number of sublayers was not attempted for the entire wavelength range due to calculation time constraints. On the level of |Δ*J*_SC_| (Figure 6c), we can observe an increasing error with respect to increasing period for the same number of sublayers and modes. By doubling the texture period (e.g., from *P* = 900 nm to 1800 nm) we need to increase the number of sublayers by more than a factor of two to achieve comparable accuracy. On the other hand, there is no significant increase in the error with respect to the number of required modes. This trend suggests that the *P* = 5000 nm result would require at least roughly 500 sublayers with ten modes for suitable accuracy (excluding artefacts), and above 1000 sublayers and 15 modes for high-accuracy simulation of such structures.

The presented analysis shows that the RCWA method can be efficiently applied for the simulation of structures with textures up to 2–3 μm periods (on a typical current desktop computer with four cores, eight threads at 4 GHz, 32 GB RAM, though presented simulations required only about 8 GB) for wide-wavelength-range simulations (350–1200 nm at a step of 10 nm). We also have to keep in mind that we simulated only a partial structure of the solar cell. Larger textures and structures of complete devices (including rear textures) would need a large number of sublayers and modes. Faster computers or clusters would enable simulation of structures larger than those presented, but still, it needs to be emphasized that doubling of both the total modes and sublayers requires a computer approximately ten times faster to solve the problem in the same time [38], ultimately limiting the size of the texture that can be considered. Larger structures such as the (pseudo-)random micropyramids introduced in Figure 4 cannot reasonably be attempted, even on current and upcoming supercomputers, and thus require additional simulation approaches that can accurately deal with large sizes.

### Application of the simulation tools

#### Optimization of the nanopyramids with RCWA

We performed optimization of the inverted nanopyramids by means of RCWA simulations. In particular, the PF was varied from the starting value of 0.7 (as depicted in Figure 4) to 1. The PF was found to be an important parameter for optimization of the antireflection effect. In this optimization, the period of the unit cell was held at 900 nm. The depth of the pyramid was changed accordingly with the PF, maintaining the same angle of the pyramid facets (linked to anisotropic etching), while the thickness of ITO and a-Si:H was kept constant.

**Figure 7:**
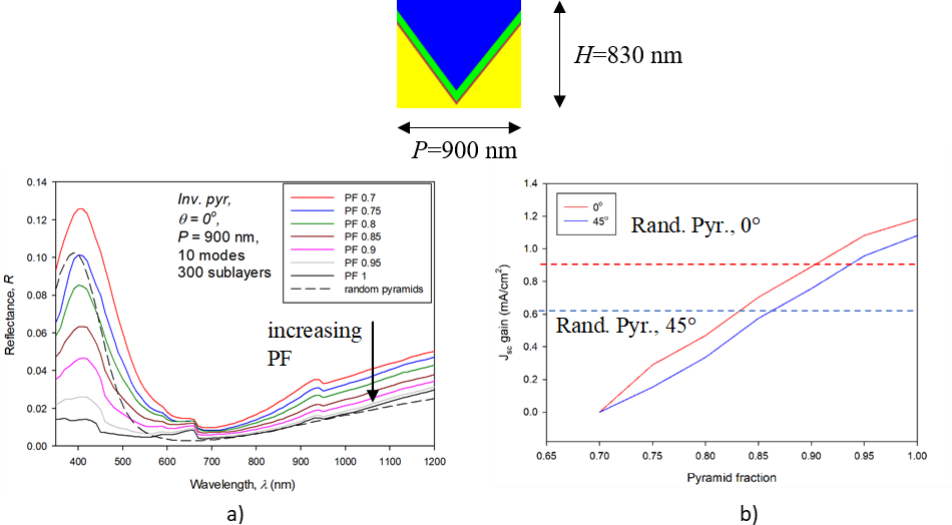
The effect of increasing the pyramid fraction (PF) in the texture in the front part of the solar cell. Reflectance as a function of wavelength for normal incident light (a) and the resulting *J*_SC_ gain for the two incident angles (b).

In Figure 7 the effects of PF variation on reflectance and on the corresponding *J*_SC_ gain are shown. The front part of the solar cell was simulated by RCWA as in previous sections. In these simulations, ten modes and 300 sublayers were used. We optimized the PF with respect to decreased reflectance of the front part of the cell, giving the possibility to additionally increase *J*_SC_. The presented *J*_SC_ gain shows the full potential of the improved antireflection effect, where all additionally in-coupled light would be absorbed and transferred into photocurrent. Thus, these are the maximal potential gains related to the given PF variation. As a reference (zero gain) the structure with PF 0.7 was taken. In Figure 7a the reflectance curves are shown for the case of normal incident light. As a reference, the reflectance curve corresponding to the random micropyramid texture is added to the graph (calculated by RT/TMM). The results indicated that by increasing the PF of the inverted nanopyramids, the antireflection effect is improved (reflectance curves decrease monotonically). In the short-wavelength region (λ < 600 nm) the textures with an increased pyramid fraction (PF > 0.75) exhibit smaller reflection than the typically used random microtextures. However, at longer wavelengths, only the curve corresponding to the highest pyramid fraction (PF 1) approaches the results of the random pyramid case.

In Figure 7b the corresponding *J*_SC_ gains are shown for the case of normal (0°) and non-normal (45°) incident light. In both cases, the *J*_SC_ gain increases monotonically while increasing the PF. The zero-gain case is set to the structure with PF 0.7. The reference lines corresponding to the random microtexture are added for the two incident angles. The results of the optimization show the potential for a *J*_SC_ gain of 1.18 mA/cm^2^ and 1.08 mA/cm^2^ for normal incident light and 45° incident illumination, respectively. Additional simulations were performed to investigate the effect of the period of the inverted pyramid texture and showed that increasing the *P* from 900 nm to 1800 nm with PF 0.7 leads to further (minor) improvement; however, the difference is just 0.1 mA/cm^2^.

#### Simulations of an encapsulated solar cell with textures

**Figure 8:**
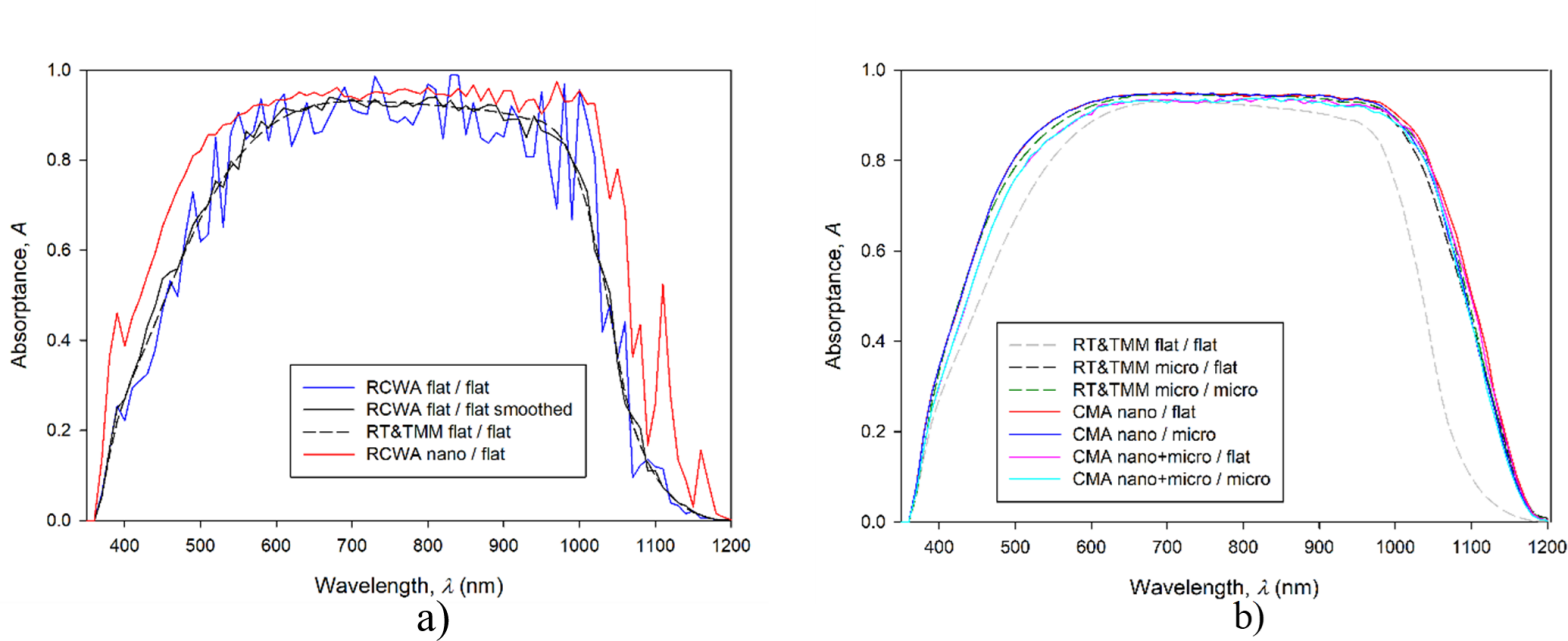
Simulated absorptances in the c-Si layer of the HJ Si solar cell using RCWA, RT/TMM (CROWM simulator) and CMA. Comparison between RCWA and RT/TMM is shown in (a). (b) RT/TMM and CMA simulations of the solar cell with a nanotexture with PF 1. Corresponding *J*_SC_ values calculated from the absorptance curves are listed in Table 2.

In the following, we present simulation results of complete HJ Si solar cell (structure from Figure 3) with different textures (as indicated in Figure 4) applied to the front and to the rear side of the solar cell. We are using the optimized nanotexture from the preceding section (*P* = 900 nm, *D* = 730 nm, PF = 1) in all graphs presented in Figure 8. Incident light is at 0°. Simulation parameters are summarized in Table 1.

In Figure 8 simulated absorptance curves (*A*) in the c-Si wafer of the encapsulated HJ Si solar cell are presented for various textures applied to the front and/or rear part of the solar cell. Different simulation approaches were applied. We assumed an ideal extraction of light-generated charge carriers from the c-Si wafer and neglected contributions of carriers from thin amorphous layers (replicating state-of-the-art devices). Under this realistic assumption, the *A* can be assumed to be equal to the external quantum efficiency, EQE, of the device [18]. In this case, the potential *J*_SC_ of the solar cells can be calculated directly from *A* by applying the AM1.5g solar spectrum (*S*) with the following equation (see *J*_SC_ values in Table 2):

[2]
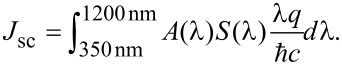


**Table 2:** Simulated *J*_SC_ of the encapsulated solar cells with different textures.

simulation method	front texture/rear texture	*J*_SC_ (mA/cm^2^)	relative *J*_SC_ gain (%)

RT/TMM	flat/flat	32.96	0
CMA	nano+micro/micro	36.24	10.0
CMA	nano+micro/flat	36.43	10.5
RT/TMM	micro/flat	36.69	11.3
RT/TMM	micro/micro	36.88	11.9
CMA	nano/micro	37.11	12.6
CMA	nano/flat	37.39	13.4

We additionally assume that, due to the same thickness of the ITO and a-Si:H layers, the electrical performance should be similar for all simulated structures.

In Figure 8a we show the results of the RCWA and RT/TMM simulations applied to a complete HJ Si solar cell structure with (i) flat front and flat rear interfaces (denoted by flat/flat) and (ii) nanotextured front (inverted nanopyramids) and flat rear interfaces (nano/flat). In RCWA simulations of the complete device, interference fringes are observed. They originate from the fully coherent treatment of the thick c-Si wafer in RCWA and are not present in experimental spectral measurements of solar cells (not shown here). The interferences can be smoothed out by averaging the simulation results, especially if simulated at 1 nm wavelength accuracy. The smoother result shown in the figure was achieved by averaging the absorption at 11 wavelengths simulated on 1 nm and presented on 10 nm, as with all other simulations. The convolution of the absorption with a Gaussian function produces similar results to this averaging (not shown). This averaged curve is in good agreement with the result of the same (flat/flat) structure simulated by the RT/TMM tool, although the RT/TMM simulation results are still smoother.

The simulation of the structure in nano/flat configuration simulated by RCWA is shown only for the non-averaged case. Even this non-smoothed curve indicates the improvement trend in *A* when a nanotexture is introduced at the front. The improvements are observed in short- and long-wavelength regions of *A*. The short-wavelength range improvement is a consequence of better antireflection (AR) properties at the front interfaces, compared to the flat structure in this case. For wavelengths in the range 500–800 nm, the optimized front thin film stack serves as an efficient AR coating already in the flat device, so the addition of nanotexture does not improve the results much further in this wavelength region. The differences in the long-wavelength region of *A* are a consequence of light scattering on the front nanotexture, enhancing the light trapping effect in the structure. With the results shown in Figure 8a, we exploit the potential of efficient simulation of the cell with RCWA and proceed with the CMA, which opens possibilities for a broad range of textures and their combinations.

Figure 8b presents the results of CMA simulations for various combinations of front and rear textures. To indicate the improvements in *A* related to different textures and their combinations, a reference curve corresponding to the RT/TMM simulation of the flat/flat cell is shown also in this figure (grey curve). In all simulations shown in Figure 8b no interference fringes are observed since in the RT/TMM and CMA methods, thick layers are treated incoherently. Whereas the RT/TMM method itself is applied to the structures (including flat interfaces and microtextures), the CMA is used for all structures, including nanotextures. Next, we focus on the improvements in solar cell performance related to the different textures and their combinations. The first observation is that in the wavelength region 650–750 nm, all *A* curves are relatively close together as the selected thicknesses of the front thin layers assure good AR properties (already in the flat/flat case). However, significant improvements related to the textures are observed in short- and especially in long-wavelength region. According to the optimization results obtained in the section “Optimization of the nanopyramids with RCWA” for the front part of the structure, the cells with a nanotextured front interfaces (nano/flat, nano/micro) exhibit the highest gain in *A* in the short-wavelength part (λ < 600 nm). In this wavelength region, the rear part of the cell does not influence *A* as the light is absorbed before reaching there. In the long-wavelength region, the simulation showed that the total increase in the optical path due to light scattering or refraction of long-wavelength light is mainly caused by increased back reflections at the front interfaces of the devices. By internal redirection of light propagation, total reflection of light waves can occur at front interfaces if the incident angles meet the condition of total reflection. The nano/flat combination of textures performed the best, while the second-best nano/microtexture had comparable light trapping ability, but higher parasitic absorption in the textured rear layers.

Among the combinations tested, special attention should be paid to the combined nano + micro front texture. From the simulation results, it can be observed that such a combined texture surprisingly does not outperform the solar cells with (single) nano- or microtextures on the front. Its main drawback was poor AR performance in the short-wavelength region. However, it is known that, in general, combined textures have potential to outperform the corresponding individual textures [4], but as indicated from the results, they must be carefully optimized for the particular solar cell structure. Here simulations can play an important role.

The improvements in *A* shown in Figure 8b were also transferred to the *J*_SC_ values of the complete solar cells. The absolute *J*_SC_ values and their relative improvement to the flat/flat case are summarized in Table 2. The simulated results are listed from lowest to highest *J*_SC_.

Table 2 shows simulated *J*_SC_ values in ascending order. All textures significantly improved the *J*_SC_ of the solar cell by at least 10% compared to the flat case. The nano/flat texture performs the best, in accordance with the observed *A* trends in Figure 8b, reaching *J*_SC_ = 37.39 mA/cm^2^. In addition to the *J*_SC_ being 4.43 mA/cm^2^ (13.4%) higher than that of the untextured solar cell, it also outperforms the microtextured solar cell by 0.51 mA/cm^2^ (1.4%). This is significantly higher than the accuracy of the CMA, estimated at 0.1 mA/cm^2^. At the solar cell level, further improvement in *J*_SC_ might be achieved for example by optimizing the thin-layer thickness for the particular texture. Furthermore, the optimization of the solar cell or photovoltaic module structure might include additional antireflective and light management coatings.

## Conclusion

In the paper, we have analyzed the RCWA performance in terms of reliability and accuracy of the optical simulation of solar cell structures, in particular for HJ Si solar cells with textures for light management. For efficient simulation of whole HJ Si solar cells, including nano- and microtextures, a coupled modeling approach (CMA) was introduced where RCWA is coupled with RT/TMM. We tested the applicability and accuracy of RCWA by changing the number of sublayers and modes in RCWA simulations. The analysis showed that RCWA is an efficient simulation method for structures with textures in the nanometer size range. RCWA was applied to optimize inverted nanopyramid textures at the front side of a HJ Si solar cell by changing the pyramid fraction (PF). The optimized nanotexture had a PF of 1, i.e., full pyramid coverage.

We applied the CMA to simulate the complete HJ Si solar cell structure, including the front encapsulation and glass, including nano- and microtextures at the interfaces of the cell. The simulation results showed that optimized nanotextures can outperform currently used microtextures, resulting in a *J*_SC_ increase of 0.51 mA/cm2 (1.4%). A combined nano/microtexture was shown to require further individual optimization as the simulations currently indicate it does not outperform the front nano- or microtexture individually.
